# Improving breast cancer prediction using a pattern recognition network with optimal feature subsets

**DOI:** 10.3325/cmj.2021.62.480

**Published:** 2021-10

**Authors:** Serdar Gündoğdu

**Affiliations:** Department of Computer Technologies, Dokuz Eylül University, Izmir, Turkey

## Abstract

**Aim:**

To predict the presence of breast cancer by using a pattern recognition network with optimal features based on routine blood analysis parameters and anthropometric data.

**Methods:**

Sensitivity, specificity, accuracy, Matthews correlation coefficient (MCC), and Fowlkes-Mallows (FM) index of each model were calculated. Glucose, insulin, age, homeostatic model assessment, leptin, body mass index (BMI), resistin, adiponectin, and monocyte chemoattractant protein-1 were used as predictors.

**Results:**

Pattern recognition network distinguished patients with breast cancer disease from healthy people. The best classification performance was obtained by using BMI, age, glucose, resistin, and adiponectin, and in a model with two hidden layers with 11 and 100 neurons in the neural network. The accuracy, sensitivity, specificity, FM index, and MCC values of the best model were 94.1%, 100%, 88.9%, 94.3%, and 88.9%, respectively.

**Conclusion:**

Breast cancer diagnosis was successfully predicted using only five features. A model using a pattern recognition network with optimal feature subsets proposed in this study could be used to improve the early detection of breast cancer.

Cancer is the second leading cause of death globally, with 9.6 million deaths in 2018. The most common cancers in women are breast, lung, cervical, colorectal, and thyroid cancer, while men most frequently suffer from lung, liver, stomach, colorectal, and prostate cancer (1). Survival rates of breast cancer patients worldwide vary greatly. The low survival in underdeveloped countries can mainly be explained by a lack of early detection systems equipped with advanced technologies (2). A lower risk of dying from breast cancer is directly related to an earlier treatment (3). Therefore, an early diagnosis, necessary to increase the survival rate of breast cancer patients, continues to be the most significant component of breast cancer control (4).

Several biomarker candidates for breast cancer have been reported in the literature (5), as well as different biomarker combinations (6-10). A combination of BMI, leptin levels, leptin/adiponectin ratio, and CA 15-3 levels as biomarkers for breast cancer has shown high reliability (9). Routine blood analyses, leptin, adiponectin, especially insulin, glucose, resistin, homeostatic model assessment (HOMA), monocyte chemoattractant protein-1 (MCP-1), age, and body mass index (BMI) data can also be used to diagnose breast cancer (10).

Data-mining classification methods can aid in the diagnostic process due to their accuracy and rapidity (11). Hwa et al (6) have reported 85% predictive sensitivity for the classification of breast cancer using a software tool with a logistic regression model. In 2015, the relationship between serum irisin levels and breast cancer was analyzed using logistic regression analysis. Serum irisin levels were found to discriminate breast cancer patients with 91.1% specificity and 62.7% sensitivity (12). Patrício et al (10) used a support vector machine (SVM) for breast cancer prediction. The sensitivity and specificity values were in the range of 82%-88% and 85%-90%, respectively. Using K-nearest neighbor (KNN) and SVM algorithms, Gündoğdu (13) predicted breast cancer risk with 85.3% accuracy, 80.8% sensitivity, and 89.1% specificity.

Pattern recognition networks (PRN) are artificial neural networks (ANNs) that are widely used to solve the classification problem (14), especially in the medical sciences. ANN models have been frequently used in cancer classification (15) and other areas of bioinformatics (16-19). Saritas and Yaşar (20) classified breast cancer with an accuracy of 86.95% when using ANN and with an accuracy of 83.54% when using Naïve Bayes algorithms (20).

The aim of this study was to predict the risk for breast cancer by using a PRN with an optimal feature set, including the routinely collected blood analysis parameters and anthropometric data. A secondary aim was to improve the classification performances, including accuracy, sensitivity, specificity, Matthews correlation coefficient (MCC), and Fowlkes Mallows (FM) index, and to create a machine learning-based model that can help physicians in the early diagnosis of breast cancer.

## Material and methods

**Figure 1:**
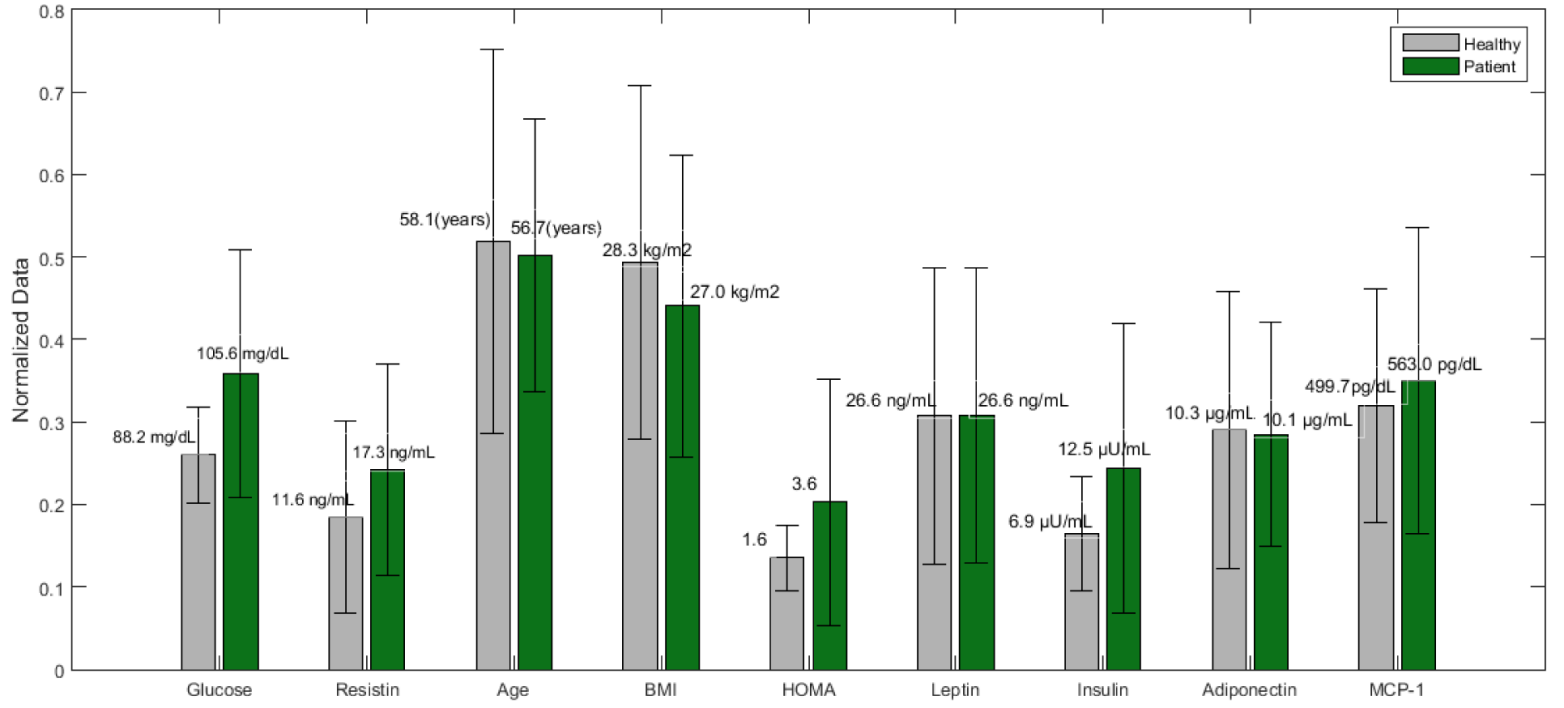
Blood analysis parameters and anthropometric data (mean and standard deviation) of 52 healthy controls and 64 patients with breast cancer

Recently, computer-generated diagnostic systems have been widely applied to detect different types of abnormalities (21). This study used breast cancer data by Patrício et al (10). The data set (in CSV format) consisted of data for 116 participants: 52 healthy participants and 64 breast cancer patients. Data on participants' age, HOMA, leptin, adiponectin, BMI, insulin, glucose, resistin, and MCP-1 were available (Figure 1). The data set was not preprocessed.

### Pattern recognition networks

Pattern recognition refers to the automated recognition of patterns and regularities in data. It is closely related to machine learning and artificial intelligence. Pattern recognition networks (patternnet) are feedforward neural networks (FFNN) that can be trained to classify inputs according to target classes (22) and are widely used in classification problem solving, especially in medical sciences (14). FFNN was the earliest kind of ANN (23,24). *Patternnet* function returns a pattern recognition neural network with a hidden layer, a training function, and a performance function.

ANNs simulate the structure of biological neural networks, with basic building blocks being artificial nerve cells. The value of a neuron is obtained by multiplying the value of each neuron in the previous layer by the weights and by adding these multiplication operations. A neural network consists of one or more hidden layers, as well as of an input and an output layer.

In the FFNN method, the data that came from the hidden layer (HL) to the j^th^ neuron and the output layer to the k^th^ neuron are calculated using the Equations 1-2, respectively (25).


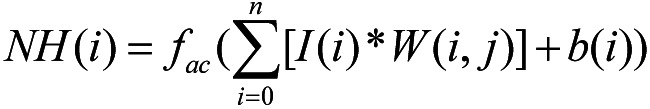
1

where *NH(i)* is the neuron in HL, *I(i)* is the inputs, *W(i, j)* is the vector of weights, *b(i)* is the bias weight, and f_ac_ is the activation function.


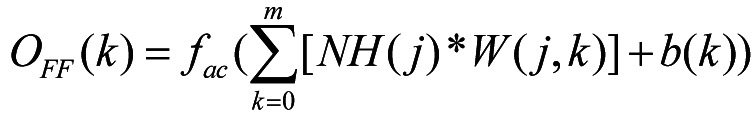
2

where *O_FF_ (k)* is the output neuron, *NH(j)* is the hidden neuron, *W(j,k)* is the vector of weights, *b(k)* is the bias weight, and f_ac_ is the function.

The breast cancer data samples were randomly divided so that 70% was used for training, 15% for validation, and 15% for testing. Different three-layer feed-forward neural networks were developed by creating different combinations of input and hidden nodes (Table 1).

**Table 1:** Hyperparameters of the selected pattern recognition networks models*

PRN parameters	M1	M2	M3	M4	M5	M5-9	M5-4
Number of variables in input layer	5	5	5	5	5	9	4
Number of the HL	1	1	1	1	2	2	2
Number of neurons in HL1	1	10	11	100	11	11	11
Number of neurons in HL2	-	-	-	-	100	100	100
Data division	Rm	Rm	Rm	Rm	Rm	Rm	Rm
Training function	LM	LM	LM	LM	LM	LM	LM
Activation function	T-S	T-S	T-S	T-S	T-S	T-S	T-S
Gradient	1E-7	1E-7	1E-7	1E-7	1E-7	1E-7	1E-7
Validation checks	6	6	6	6	6	6	6

*Abbreviations: M – model; Rm – random; LM – Levenberg-Marquardt; T-S – tan-sigmoid; HL – hidden layer.

The model training function, activation function, and loss function were Levenberg-Marquardt algorithm, tan-sigmoid, and mean square error (MSE), respectively. The number of iterative learning steps (epochs) was 1000. The validation check number in the neural network training was 6.

According to Du and Stephanus (26), the LMA performed significantly better than other training algorithms (26). MSE is the sum of squared distances between the observed and predicted values, which is the most commonly used loss function (27). Ullah et al (28) used the tan-sigmoid as the activation function. They also claimed that MSE might be the best parameter to find the best activation function (28).

The number of hidden layers and neurons greatly affects the network performance. There is no methodology for the selection of hidden layers or neurons so trial-and-error method is often used for this purpose (29). To find the best performance, several neural network models were created with different hidden layers and neurons.

This study used a deep learning toolbox (MATLAB, MathWorks, Natick, MA, USA, release 2020a), which provides a framework for designing and implementing deep neural networks.

A confusion matrix was used to compare the models' performance by evaluating the classification accuracy. The matrix has four components: true negatives (TN), true positives (TP), false negatives (FN), and false positives (FP) (Table 2). Healthy and patient-labeled samples were considered as a positive class and negative class, respectively. TP are the samples of healthy participants correctly classified as healthy, FP are the samples of patients classified as healthy, TN are the samples of patients classified as diseased, and FN are the samples of healthy participants classified as diseased.

**Table 2:** Confusion matrix for binary classification

	Target class		
Output class	Healthy	Patient	Total
Healthy	**TP**	FP	TP+FP
Patient	FN	**TN**	FN+TN
Total	TP+FN	FP+TN	

*Abbreviations: TN – true negatives; TP – true positives; FN – false negatives; and FP – false positives.

Accuracy, sensitivity, specificity, MCC, and FM index from a confusion matrix were used to evaluate the performance of the classifier models. These scores were defined as shown in Equations 3-7.


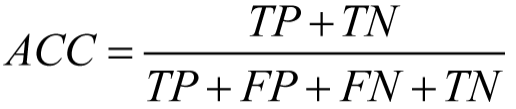
3


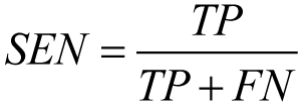
4


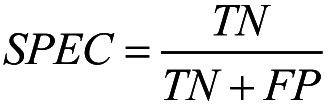
5


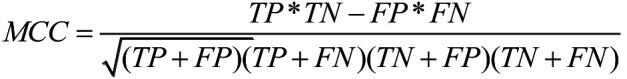
6


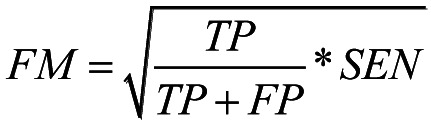
7

## Results

**Figure 2:**
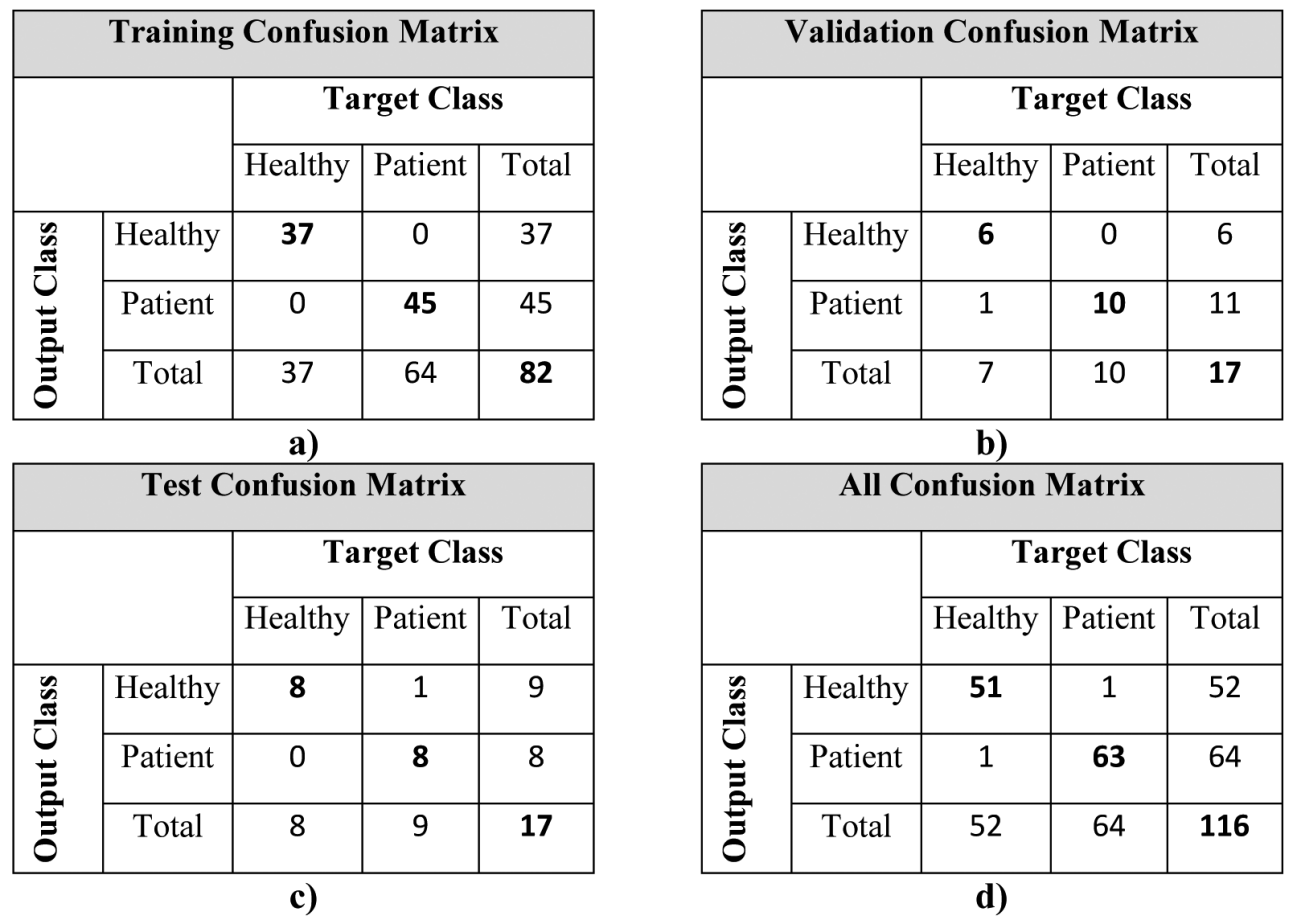
Pattern recognition network confusion matrix for the best results from the network’s testing (**A**), training (**B**), validation (**C**), and all (**D**) data.

In M1, M2, M3, M4, and M5, age, BMI, glucose, resistin, and adiponectin were used as input. In the models, there were 1, 10, 11, 100, and 11-100 (two hidden layers) neurons in the hidden layers, respectively. Confusion matrices were created for each model from simulation results. The confusion matrix of the results of M5, the most successful model, is shown in Figure 2.

The M5 correctly identified 114 out of 116 samples. By applying Equations 3-7 to the confusion matrices obtained using M1, M2, M3, M4, and M5, we calculated sensitivity, specificity, accuracy, MCC, and FM index values (Table 3).

**Table 3:** Classification performance results of the neural network with five features, according to the number of hidden layers (HL) and the number of neurons in the HL

Models		Accuracy	Sensitivity	Specificity	Fowlkes-Mallows index	Matthews correlation coefficient
**M1** (HL-**1**) (HL1-**1**)	training	79.3	77.8	80.4	76.7	58.1
	validation	82.4	66.7	100.0	81.6	69.6
	test	70.6	71.4	70.0	66.8	40.8
	all	78.4	75.0	81.3	75.7	56.4
**M2** (HL-**1**) (HL1-**10**)	training	87.8	91.7	84.8	87.0	75.9
	validation	94.1	100.0	90.0	93.5	88.7
	test	70.6	88.9	50.0	77.0	42.6
	all	86.2	92.3	81.3	85.9	73.2
**M3** (HL-**1**) (HL1-**11**)	training	93.9	91.9	95.6	93.2	87.7
	validation	82.4	71.4	90.0	77.2	63.2
	test	82.4	75.0	88.9	80.2	64.8
	all	90.5	86.5	93.8	89.1	80.8
**M4** (HL-**1**) (HL1-**100**)	training	100.0	100.0	100.0	100.0	100.0
	validation	94.1	80.0	100.0	89.4	85.9
	test	82.4	85.7	80.0	80.2	64.8
	all	96.6	96.2	96.9	96.2	93.0
**M5** (HL-**2**) (HL1-**11**) (HL2-**100**)	training	100.0	100.0	100.0	100	100
	validation	94.1	85.7	100.0	92.6	88.3
	test	94.1	100.0	88.9	94.3	88.9
	all	98.3	98.1	98.4	98.1	96.5

The M1 yielded the worst results, with accuracy, sensitivity, specificity, FM index, and MCC of 70.6%, 71.4%, 70%, and 66.8%, respectively. The MCC calculated for the test confusion matrix was 40.8%.

The best results were obtained with the M5 model, which had 11-100 neurons in the hidden layer 1 and hidden layer 2, respectively. The accuracy, sensitivity, specificity, MCC, and FM index from the test confusion matrices were 94.1%, 100%, 88.9%, 94.3%, and 88.9%, respectively, which shows the effectiveness of this model.

**Figure 3:**
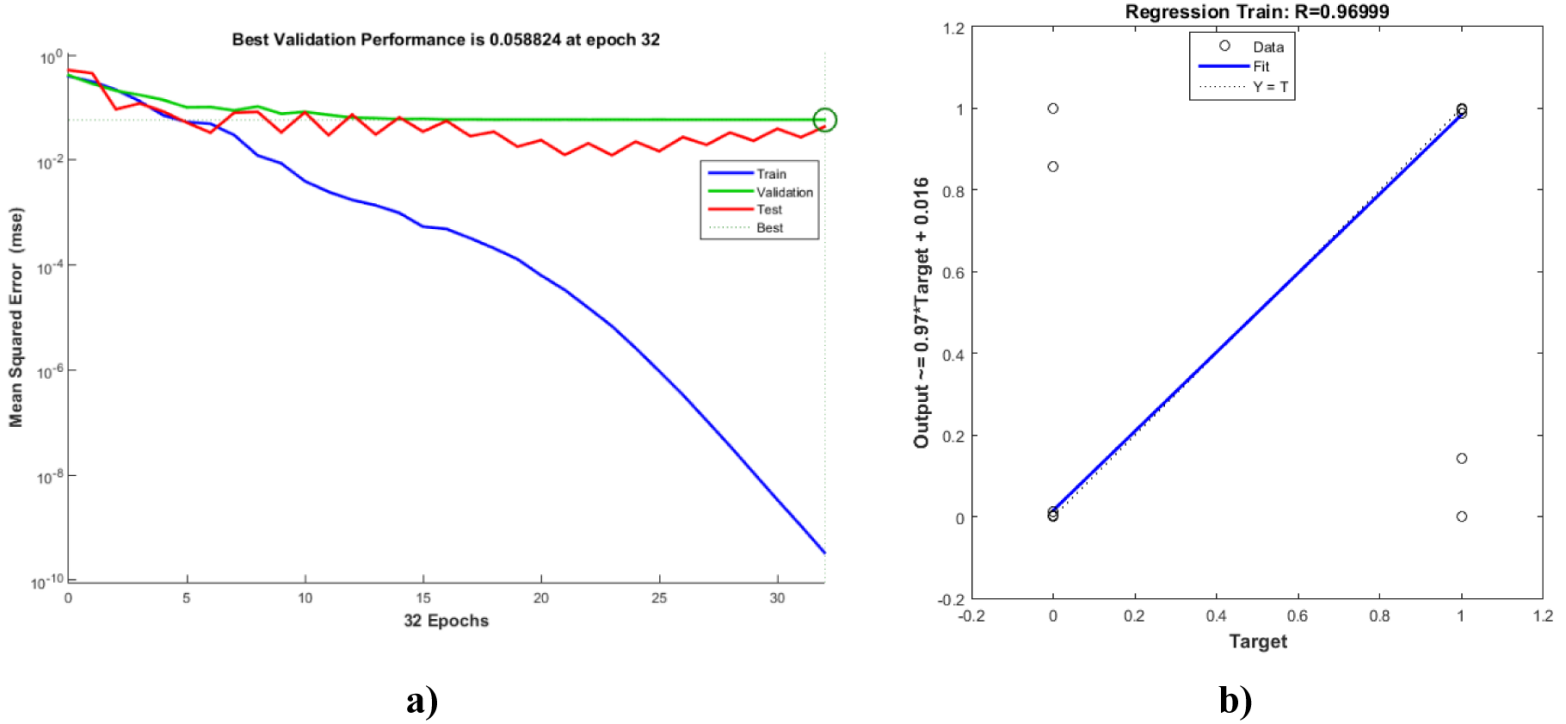
(**A**) Best validation and (**B**) regression performance of the model 5 for the presence of breast cancer.

In the neural network training, the epoch and validation checks numbers were 1000 and 6, respectively. The epoch vs MSE variations that occurred during the training phase are shown in Figure 3A. The MSE decreased with the increase in the number of epochs for all training, validation, and test data. The trend slope was decreased when a fixed error started to persist for the network model. The best validation performance in terms of MSE was 0.058824 at epoch 32. The R^2^ value, which shows the relationship between the actual data and the data predicted by the PRN, was approximately 0.941 (Figure 3B). This result shows the high simulation capability of the model used.

**Figure 4:**
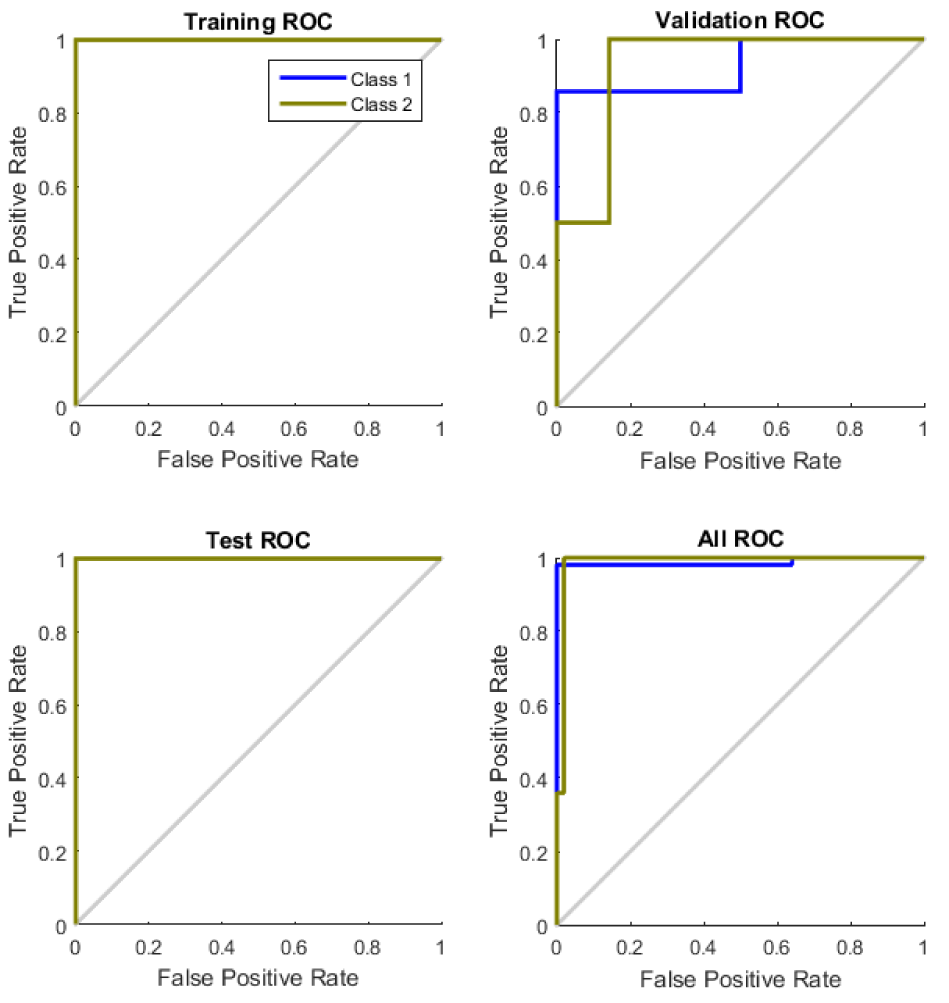
Pattern recognition network’s corresponding area under the curve (AUC) for the best results from the network’s (**A**) training; (**B**) validation; (**C**) test; and (**D**) all data. ROC: receiver operating characteristic.

The classification results also corresponded with the area under the curve (AUC) for each confusion matrix of the M5 model (Figure 4).

To compare the results of this study with two studies (10,20) using the same data, 9 (including age, HOMA, leptin, adiponectin, body mass index, insulin, glucose, resistin, and MCP-1) and 4 features (resistin, glucose, age, and BMI) were entered in the M5 instead of 5 features (Table 4).

**Table 4:** Classification performance results of the neural network with 9 and 4 predictors according to the number of hidden layers (HL) and the number of neurons in the HL*

Models	Number of features		Accuracy	Sensitivity	Specificity	Fowlkes-Mallows index	Matthews correlation coefficient
**M5-9** (HL-2) (HL1-11) (HL2-100)	9	training	80.5	75.0	84.8	77.2	60.2
		validation	82.4	85.7	80.0	80.2	64.8
		test	70.6	77.8	62.5	73.8	40.9
		all	79.3	76.9	81.3	76.9.	58.2
**M5-4** (HL-2) (HL1-11) (HL2-100)	4	training	90.2	86.1	93.5	88.6	80.2
		validation	88.2	100.0	80.0	88.2	78.9
		test	82.4	77.8	87.5	82.5	65.3
		all	88.8	86.5	90.6	87.4	77.3

## Discussion

In this study, the results obtained by M5 with 5 features (age, BMI, glucose, resistin, and adiponectin) showed that pattern recognition networks can be effectively used for breast cancer prediction.

Compared with the previous studies, the results of the M5 model in this study may be considered reliable and highly accurate. Sarıtaş and Yaşar (20) compared the performance results of ANN and naïve Bayes classifiers applied to data with the same 9 clinical features. The data samples were selected so that 65% were used for training, 25% for testing, and 10% for validation. Breast cancer was classified with an accuracy of 86.95% when ANN were used and with an accuracy of 83.54% when naïve Bayes algorithms were used. In the current study, while the classification accuracy for the M5-9 model with 9 features was 70.6%, the accuracy of the M5 model with 5 features was 94.1%.

Patrício et al (10) predicted the presence of breast cancer in women based on 4 features (age, resistin, BMI, and glucose) in the same 9-feature data set, with a specificity ranging from 85% to 90% and a sensitivity ranging from 82% to 88%. In the current study, while the specificity and sensitivity values for the M5-4 model with 4 features were 87.5% and 77.8%, respectively, for the M5 model with 5 features they were 88.9% and 100%.

Gündoğdu (13) used KNN and SVM algorithms with 5 features (age, BMI, glucose, resistin, and adiponectin) as inputs for the prediction of breast cancer. This model had 85.3% accuracy, 80.8% sensitivity, and 89.1% specificity. The results of the M5 model in this study showed that the predictions of the PRN were better than the predictions of KNN and SVM methods, with 94.1% accuracy, 100% of sensitivity, and 88.9% of specificity.

This study proposed a model for predicting the presence of breast cancer by using the PRN with relevant optimal attributes. The best classification was obtained when age, BMI, glucose, resistin, and adiponectin were applied as network inputs. These results could be used to aid physicians in the detection of breast cancer. Although it is a matter of discussion whether breast cancer estimation can be used instead of imaging techniques, detecting the disease with routine blood analysis parameters and anthropometric data may cause less stress, anxiety, and pain for the patients.
